# Microbiota–Gut–Brain Axis and Epilepsy: A Review on Mechanisms and Potential Therapeutics

**DOI:** 10.3389/fimmu.2021.742449

**Published:** 2021-10-11

**Authors:** Manqiu Ding, Yue Lang, Hang Shu, Jie Shao, Li Cui

**Affiliations:** Department of Neurology, First Hospital of Jilin University, Changchun, China

**Keywords:** epilepsy, gut–brain axis, microbiota, pathogenesis, therapy

## Abstract

The gut–brain axis refers to the bidirectional communication between the gut and brain, and regulates intestinal homeostasis and the central nervous system *via* neural networks and neuroendocrine, immune, and inflammatory pathways. The development of sequencing technology has evidenced the key regulatory role of the gut microbiota in several neurological disorders, including Parkinson’s disease, Alzheimer’s disease, and multiple sclerosis. Epilepsy is a complex disease with multiple risk factors that affect more than 50 million people worldwide; nearly 30% of patients with epilepsy cannot be controlled with drugs. Interestingly, patients with inflammatory bowel disease are more susceptible to epilepsy, and a ketogenic diet is an effective treatment for patients with intractable epilepsy. Based on these clinical facts, the role of the microbiome and the gut–brain axis in epilepsy cannot be ignored. In this review, we discuss the relationship between the gut microbiota and epilepsy, summarize the possible pathogenic mechanisms of epilepsy from the perspective of the microbiota gut–brain axis, and discuss novel therapies targeting the gut microbiota. A better understanding of the role of the microbiota in the gut–brain axis, especially the intestinal one, would help investigate the mechanism, diagnosis, prognosis evaluation, and treatment of intractable epilepsy.

## 1 Introduction

Epilepsy is a chronic neurological disorder that affects >70 million people worldwide (1) with a considerable social and economic burden. Characterized by relapses and unprovoked spontaneous seizures (2), its mechanisms are complicated, and 60% of cases are idiopathic (3). In clinical practice, a diagnosis of epilepsy is challenging. If patients have infrequent seizures, the electrical markers for diagnosis may not be present, and epileptiform discharges may occasionally occur in patients who do not have seizures. The most common antiepileptic treatments are pharmaceutical, including low-cost medications and new drugs. However, in >30% of patients with epilepsy, seizures cannot be controlled with drug therapy, a phenomenon known as refractory epilepsy (4). The *ad hoc* Task Force of the International League Against Epilepsy (ILAE) defined drug resistance as “failure of adequate trials of two tolerated, appropriately chosen and used antiepileptic drug schedules (whether as monotherapies or in combination) to achieve sustained seizure freedom” (5). Despite alternative treatments such as dietary control, nerve stimulation, and surgery, still some patients do not improve. Although the effectiveness of seizure focus resection is high, not all patients benefit from it, some exhibit obvious adverse effects, and surgery alone may not be sufficient, due to the complex etiology of epilepsy (6, 7). Therefore, there is a need to develop more effective protocols for the diagnosis and treatment of epilepsy.

**Figure 1:**
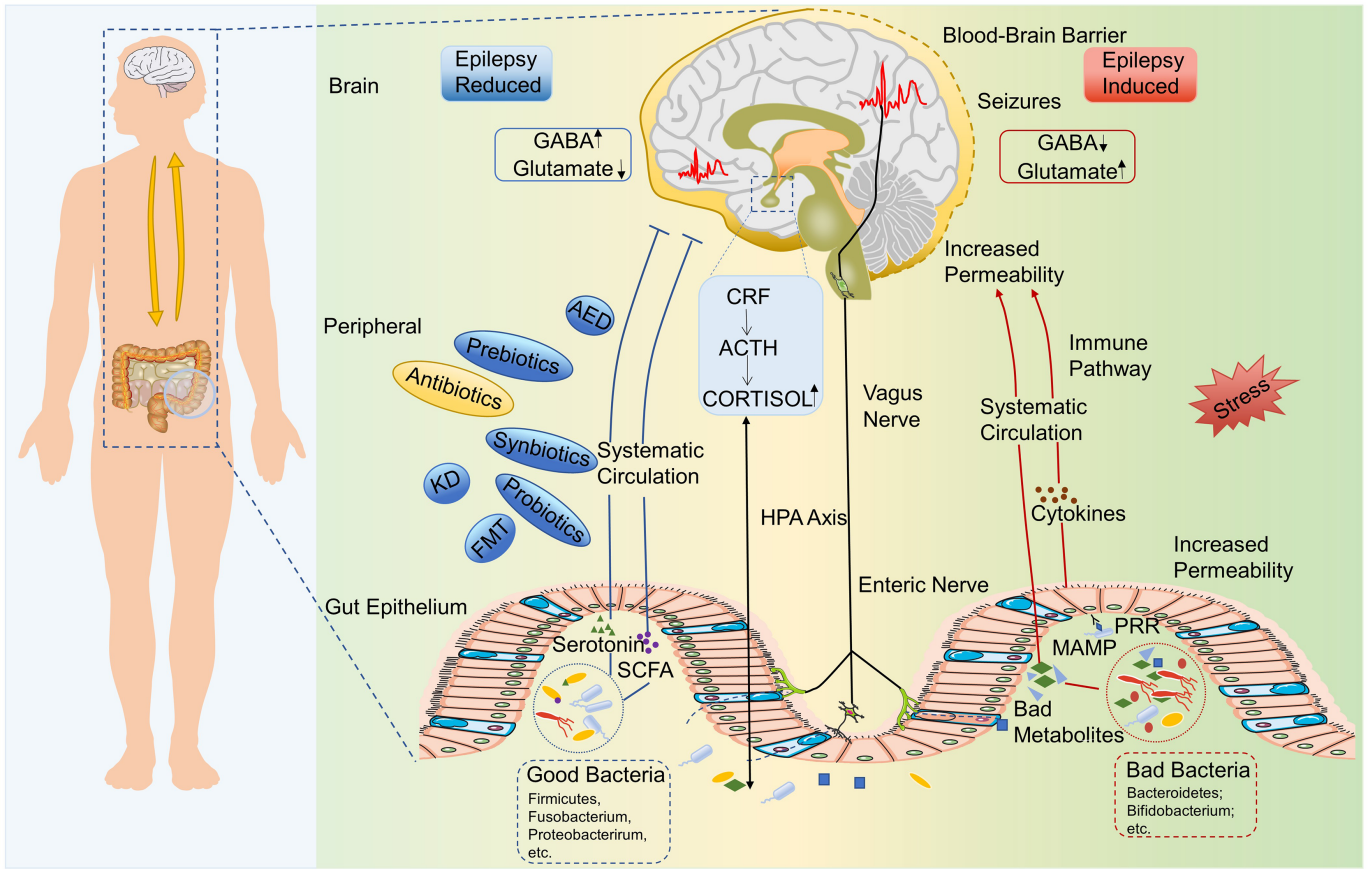
The microbiota–gut–brain axis in epilepsy. Bad gut microbiota could upregulate the production of epilepsy-promoting metabolites, the secretion of inflammatory factors, and so on, which lead to abnormal GABA/glutamate ratio and then induce the epilepsy. Chronic stress may be a trigger for this process. Healthy gut microbiota could produce good metabolites, such as SCFAs and serotonin, which could inhibit the occurrence of epilepsy. HPA axis, enteric nervous system, and vagus nervous system are also involved in the interaction between gut microbiota and epilepsy. ACTH, Adrenocorticotropic hormone; AED, Antiepileptic drug; CRF, Corticotrophin-releasing factor; HPA, Hypothalamic–pituitary–adrenal; GABA, γ-aminobutyric acid; KD, Ketogenic diet; MAMP, Microbe-associated molecular pattern; SCFA, Short-chain fatty acid; PRR, Pattern recognition receptor.

Epileptic patients often have gastrointestinal symptoms, while patients with inflammatory bowel disease have a higher susceptibility to epilepsy (8). Ketogenic diet (KD) has been used for a long time as a non-pharmacological therapy in drug-resistant epileptic patients not suitable for surgery—especially in children—with good curative effects (9). These clinical phenomena support a relationship between the gut and epilepsy. Recent advances in sequencing technology have allowed studies on the composition and function of microbiota in neurology. In recent years, some studies have suggested statistical differences in fecal microbial composition between epileptic patients and healthy people, as well as between epileptic patients before and after KD treatment, and in animal models (10–21). The intestinal microbiota may shape brain function through a variety of pathways and systems, including the central nervous system (CNS), the hypothalamic–pituitary–adrenal (HPA) axis, immune and inflammatory systems, and neuromodulators, and could therefore also be involved in epilepsy (**Figure 1**). Remodeling intestinal microbiota through individualized diet, probiotics, antibiotics, and even fecal microbiota transplantation (FMT) may become the future standard treatment of refractory epilepsy. Herein, we review the latest knowledge on the correlation between the gut microbiota (GM) and epilepsy.

## 2 Classic Animal Models of Epilepsy

Epilepsy is a complex syndrome with a complicated etiology (structural, genetic, infectious, metabolic, immune, and unknown) and diverse clinical manifestations (1, 22). Due to its complexity, in 2017, the ILAE developed a three-level classification, including seizure types, epilepsy types, and epilepsy syndromes (22). Epilepsy can be divided into four categories: focal epilepsy, generalized epilepsy, combined generalized and focal epilepsy, and unknown epilepsy (22). According to its responsiveness to antiepileptic drugs, epilepsy can be divided into drug-sensitive and refractory epilepsy. Together, the complex etiology, clinical signs, and classification of epilepsy determines the lack of a specific animal model reflecting all of its characteristics. Classical animal models of epilepsy include the following categories. The maximal electroshock model and pentylenetetrazol (PTZ) models are classical ones that simulate acute epilepsy (23). The kindling model simulates the characteristics of progressive development and long-term recurrence through repeated electrical and chemical stimulation of the thalamus, amygdala, hippocampus, and other regions, while continuous stimulation could induce status epilepticus. The WAG/Rij rat model is used for the study of hereditary absence epilepsy (24). In addition, animal models of epilepsy with a special etiology can be prepared by microbial infection, trauma, ischemia, and hypoxia. Chemical kindling models induced by li-pilocarpine (25) or kainic acid (26, 27), amygdala electrical kindling models (28), and genetic models have been used to construct animal models of refractory epilepsy (29). These models are important for exploring the pathogenesis of intractable epilepsy and screening and identifying new antiepileptic drugs.

## 3 The Close Relationship Between Intestinal Microbiota and Epilepsy

The microbiota, a wide variety of microorganisms populating the gut, including 50 bacterial phyla, is 10 times more abundant than the somatic and germ line cells of the human body (30). Firmicutes, Bacteroidetes, Actinobacteria, Proteobacteria, Fusobacteria, Verrucomicrobia, and Cyanobacteria are the seven dominant bacterial phyla in the human gastrointestinal tract, among which Bacteroidetes and Firmicutes constitute >90% (31–33). Although the overall distribution of GM in healthy people remains constant, temporal and spatial differences still exist. GM influences human health by regulating the metabolism and the host immune response. It has been reported that the species of GM in individuals with neuropsychiatric and neurodegenerative disorders differ from those in healthy people, but there are few reports on the correlation between epilepsy and GM (34). With the wide spread of 16S/18S rDNA sequencing, recent studies have reported that individuals with refractory epilepsy show altered GM composition (34). We elaborate on the relationship between the GM and epilepsy in humans and murine and discuss the dietary intervention for epilepsy *via* modulation of GM.

### 3.1 Human Microbiota and Epilepsy

Only a few population-based studies revealed GM differences between the epilepsy group and healthy controls (HC) in a relatively limited sample size (**Table 1**) (10–15). Xie et al. compared the GM of 14 patients with refractory epilepsy and 30 HCs and found a higher GM diversity in HCs (15). At the phylum level, Bacteroidetes was the main GM in the HC group, followed by Firmicutes, while Firmicutes predominated in patients (15). At the genus level, the GM was also significantly different between these two groups (15). Peng et al. demonstrated differences in GM diversity and composition between the drug-resistant (DR), drug-sensitive (DS), and HC groups, and that epilepsy frequency and GM were correlated (12). The alpha-diversity of the DR group was higher than that of the DS and HC groups, and similar between the HC and DS groups (12). Alpha-diversity in patients with ≤4 seizures per year was similar to that of HC, while patients with >4 seizures had significantly higher alpha-diversity (12). At the phylum level, DS and HC had similar GM composition, with Bacteroidetes as the largest phylum and Firmicutes as the second (12), while in the DR group, Firmicutes was the largest group, followed by Bacteroidetes (12). At the genus level, differences between the DS and DR groups still existed (12). *Bifidobacteria* and *Lactobacillus* were lower in patients with >4 seizures per year than in patients with ≤4 seizures per year (12). Bacterial function analysis showed that glucose- and lipid-associated metabolic pathways were all downregulated in the epileptic group and ABC (ATP-binding cassette) transporter-associated metabolic pathways elevated in the DR group compared to the DS group (12). Gong et al. investigated GM structure and composition in an exploratory cohort (epilepsy patients, *n* = 55; HC, *n* = 46) and validated the GM as a biomarker for epilepsy in a validation cohort (epilepsy patients, *n* = 13; HCs, *n* = 10) (10). A much lower GM alpha-diversity was observed in patients than in HCs (10). Actinobacteria and Verrucomicrobia increased and Proteobacteria decreased at the phylum level, while at the genus level, *Prevotella_9*, *Blautia*, *and Bifidobacterium* increased in patients with epilepsy (10). They further constructed a random forest model based on the GM and verified its value as a biomarker to discriminate epilepsy from HCs or drug-resistant epilepsy (DRE) *vs.* drug-sensitive epilepsy (DSE) (10). Safak et al. performed a contrast analysis of the fecal microbiome between idiopathic focal epilepsy (*n* = 30) and the HC group (*n* = 10), finding that Proteobacteria and Fusobacteria, which could cause autoimmune diseases, were significantly higher in the idiopathic focal epilepsy group than in the HC group, and Bacteroidetes and Actinobacteria, which have a positive effect on the immune system, were significantly lower (11). This study indicated the possible roles of autoimmune mechanisms and inflammation in the etiology of epilepsy. However, they did not perform a GM diversity analysis. Lee et al. conducted a study on the GM of eight children with intractable epilepsy and 32 HCs (13). Microbiota richness was lower in the epilepsy group than in the HC group (13). Actinobacteria was higher in the epilepsy group than in HCs, whereas Bacteroidetes was lower (13). They identified 17 and 18 species of bacteria strongly related to epilepsy and the HC group, respectively (13). *Enterococcus faecium*, *Bifidobacterium longum*, and *Eggerthella lenta* were the strongest potential biomarkers in the intractable epilepsy group (13). They also suggested the ABC transporter as a functional biomarker of intractable epilepsy, consistent with the results of Peng et al. (12, 13). Forty-four adult epilepsy patients were classified into the DRE (*n* =23) and DSE groups (*n* = 21) in a 2021 study (14). Alpha- and beta-diversity analyses showed no significant differences between patients in these two groups, but GM composition differences were related to patients’ response to epileptic drugs, magnetic resonance imaging (MRI), and electroencephalogram (EEG) (14). *Bacteroides finegoldii* and *Ruminococcus_g2* were more abundant, and *negativicutes* decreased in the DRE group; *B. finegoldii* was more abundant in patients with normal MRI, and *Bifidobacterium* was more abundant in patients with normal EEG (14). Taken together, these six clinical studies evaluated GM diversity, composition, and function in patients with epilepsy, but with partially inconsistent results. All these studies indicated GM dysbiosis in patients with epilepsy, showing the potential value of GM for epilepsy diagnosis and treatment, especially in refractory epilepsy, but there are still some contradictions. Most studies indicated a higher alpha-diversity in the HC group than in the epilepsy group (10, 13–15); however, a study suggested the opposite result (12). GM changes in patients with epilepsy are not completely consistent. However, given the many variables that could affect the gut microbiome, such as differences in study design, age, diet, and living environment, efforts are needed to conduct larger sample analyses based on reasonably controlled variables.

**Table 1:** Summary of previous studies on the intestinal microbiota in patients with epilepsy (drug-resistant/drug-sensitive).

Author	Year	Type of experimental design	Patient Group	Age	Key Findings
Gong et al. (35)	2020	Cross-sectional study	Exploration Cohort (n=55 EP and *n* = 46 HC) and Validation Cohort (*n* = 13 EP and *n* = 10 HC)	EP: 26.33± 12.05 HC: 28.5 ± 4.27	HC: a typical human diversity profile EP: lower alpha diversity; Phylum:↑Actinobacteria and Verrucomicrobia;↓Proteobacteria Genus:↑*Prevotella_9*, *Blautia*, *Bifidobacterium* DRE: Phylum: ↑Actinobacteria, Verrucomicrobia, and Nitrospirae Genus:↑*Blautia*, *Bifidobacterium*, *Subdoligranulum*, *Dialister*, and *Anaerostipes*
Şafak et al. (36)	2020	Cross-sectional study	Idiopathic focal epilepsy (*n* = 30) and HC (*n* = 10)	EP: 41.3 ± 12.2 HC: 1.7± 6.8	HC:↑Firmicutes (*Blautia*, *Coprococcus*, *Faecalibacterium*, and *Ruminococcus*), Bacteroidetes (*Bacteroides* and *Parabacteroides*), Actinobacteria (*Bifidobacterium* and *Collinsellagenus*) EP:↑Proteobacteria (*Campylobacter*, *Delftia*, *Haemophilus*, *Lautropia*, *Neisseria*), Fusobacteria
Peng et al. (37)	2018	Cross-sectional study	DRE (*n* = 42), DSE (*n* = 49), HC (*n* = 65)	DRE: 28.4 ± 12.4 DSE: 5.1 ± 14.6 HC: 29.4 ± 13.8	DSE and HC: ↑Bacteroidetes; DRE group: ↓ Bacteroidetes, ↑Firmicutes, and ↑Verrucomicrobia
Lee et al. (38)	2020	Prospective study	Intractable epilepsy (*n* = 8), HC (*n* = 32)	EP: 3.49 ± 1.76	EP:↓Bacteroidetes, Proteobacteria;↑Actinobacteria ↓Microbiota richness indices Biomarkers for intractable epilepsy:↑*E. faecium*, *B. longum, E. lenta;*↑ABCT
Lee et al. (39)	2021	Prospective study	DRE (*n* = 23) *vs*. DSE (*n* = 21)	DSE: 44 ± 17.2 DRE: 41 ± 13.6	DSE:↑*Bacteroides finegoldii*, *Ruminococcus_g2* DRE:↑*Negativicutes* Alpha and beta diversities: no significant difference between the two groups Epilepsy patients with a normal EEG: ↑*Bifidobacterium* Epilepsy patients with a normal MRI: ↑*Bacteroides finegoldii*
Xie et al. (40)	2017	Prospective observational study	Refractory epilepsy (*n* = 14) *vs.* HC (*n* = 30)	EP: 1.95 ± 3.10 HC: aged up to 3 years	HC:↑Bacteroidetes,↑Actinobacteria EP:↑Proteobacteria,↑Firmicutes

DRE, drug-resistant epilepsy; DSE, drug-sensitive epilepsy; EP, patients with epilepsy; HC, health control group.

Patients age was expressed as the mean ± SD according to the normality of distribution.

### 3.2 Murine Microbiota and Epilepsy

The susceptibility to PTZ-induced epilepsy was increased in rats with 2,4,6-trinitro-benzene-sulfonic acid (TNBS)-induced colitis (41). In a mouse model of PTZ-induced seizures, intestinal inflammation increases convulsive activity and decreases the effectiveness of antiepileptic drugs. Further, alleviation of intestinal inflammation has a specific antiepileptic effect (8). In addition, a reversible inflammatory response characterized by microglial activation and an increase in tumor necrosis factor alpha (TNFα) was observed in the hippocampus of TNBS-treated rats, suggesting that gut inflammation may increase CNS excitability by inducing CNS inflammation (41); however, the underlying mechanism is still unknown. Medel-Matus et al. revealed that chronic stress can facilitate seizure development by perturbing the GM (42). GM transplantation from stress donors to sham-stressed subjects increased seizure kindling rate and duration after kindling of the basolateral amygdala, while the proconvulsant effects of chronic stress were prevented by GM transplantation from sham stress donors (42). In WAG/Rij rats, a genetic model of absence epilepsy, GM was altered with a lower Bacteroidetes/Firmicutes ratio at the age of 1 month and before the onset of epilepsy, and a further reduced Bacteroidetes/Firmicutes ratio with a large number of absence seizures was observed at 4 months (16). Short-chain fatty acids (SCFAs) are messengers between the gut and brain, and butyrate has an anti-epileptic effect in rats (43). SCFAs were reduced in WAG/Rij rats (16). Furthermore, FMT altered the number of absence seizures in rats with concomitant GM remodeling (16). This model suggests that the GM is involved in the initiation and maintenance of hereditary absence seizures. In conclusion, both human and murine studies have shown that the GM is closely related to the occurrence of multiple types of epilepsy.

### 3.3 Dietary Intervention for Epilepsy *via* Modulation of GM

Diet is a major factor in shaping GM composition (44, 45). Zmora et al. concluded in their review that nutrients in food could shape the GM in a variety of ways: (1) directly interact and regulate microorganisms, (2) indirectly by influencing host metabolism, and (3) by passively introducing microbiota (46). Researchers analyzed fecal microbiota by applying next-generation sequencing technology and classified enterotypes into *Bacteroides*, *Prevotella*, and *Enterobacteriaceae*, which could be correlated with dietary habits (47). Long-term intake of animal protein and fat was beneficial to the growth of the *Bacteroides* enterotype, while carbohydrate-enriched diet enriched *Prevotella* (48). A classic Western diet (rich in fats or proteins) results in the reduction of beneficial butyrate-producing bacteria, *Bifidobacteria*, and *Eubacterium*. Most of the carbon and energy of the GM originate from dietary fiber (49, 50). Dietary protein is an essential source of nitrogen for GM growth, but high-protein diets are related to high levels of harmful metabolites in feces, cancer, and inflammatory bowel disease (50, 51). GM composition can be adjusted by altering the proportion of dietary fiber, protein, and fat (52). Therefore, a healthy diet and lifestyle are both important in GM formation.

KD indeed has a role in reducing the frequency of seizures, especially in refractory epilepsy; however, the underlying mechanisms need to be further elucidated. The existing mechanisms mainly involve neurotransmitters, brain energy metabolism, oxidative stress, and ion channels (53, 54). KD can induce GABA synthesis by upregulating glutamic acid and inhibiting GABA degradation by altering GABA transaminase activity (55, 56). Aspartate is a known glutamate decarboxylase inhibitor whose reduction can theoretically promote GABA synthesis. Aspartate levels were reduced in astrocytes exposed to ketone bodies, and similar declines were found in the forebrain and cerebellum of mice fed with KD (57). Ketosis increases the conversion of glutamate to glutamine in astrocytes (57). Glutamine then enters the neurons, eventually converting to GABA and increasing the inhibition of neurons. Barañano et al. reported that KD could prevent neuronal overexcitation *via* changes in brain pH, directly inhibit channels, and contribute to the conversion of the stimulatory glutamate to the inhibitory GABA (58) which could be secreted by certain *Lactobacillus* and *Bifidobacterium* strains (59). KD also promotes the production of fatty acids, particularly PUFAs, which may activate peroxisome proliferator-activated receptors that regulate anti-inflammatory, antioxidant, and mitochondrial genes, leading to enhanced energy reserves, synaptic function stabilization, and hyperexcitability restriction (60).

KD induces GM alteration and it is therefore involved in this treatment of epilepsy. At present, only four clinical studies suggest that KD may play a protective role in epilepsy patients by adjusting the GM (**Table 2**) (15, 17–19). Xie et al. performed successive KD therapy for ≥1 week in 14 infants with refractory epilepsy, after which the clinical occurrence of epilepsy was largely alleviated. *Proteobacteria* and *Cronobacter* decreased, and *Prevotella* and *Bifidobacterium* significantly increased after KD treatment (15). Zhang et al. further explored the changes in GM after KD in children (*n* = 20) and linked these changes to the differential efficacy of KD treatment (17). A lower alpha-diversity of the GM was observed after 6 months of KD (17). The abundance ratio of Bacteroidetes significantly increased, while that of Firmicutes and Actinobacteria significantly decreased during KD intervention (17). Bacteroides can regulate the secretion of 6–17 interleukins in dendritic cells, which are connected with seizures and can break down dietary fiber into SFCAs, which are beneficial for patients with epilepsy (64). Several gut bacteria (*Clostridiales*, *Ruminococcaceae, Rikenellaceae*, *Lachnospiraceae*, and *Alistipes*) were enriched in the non-responsive group, which makes them potential biomarkers and therapeutic targets in patients with non-reactive epilepsy (17). Lindefeldt et al. offered 12 children with therapy-resistant epilepsy 3-month KD and observed differences in bacterial taxa and functional structures (18). Compared with baseline, fecal microbial profiles showed an approximately identical alpha-diversity after KD therapy and a relatively decreased abundance of *Bifidobacteria*, *E. rectale*, and *Dialister* and increased abundance of *E. coli* (18). Functional analysis revealed a decline in seven pathways associated with carbohydrate metabolism after KD (18). Gong et al. treated 12 drug-resistant epileptic children during 6 months with KD and observed changes in GM composition and metabolites (19). The abundance of eight epilepsy-associated genera of GM significantly changed with decreases in *Bifidobacterium*, *Akkermansia muciniphila*, *Enterococcaceae*, and *Actinomyces* and increases in *Subdoligranulum*, *Dialister*, and *Alloprevotella*, which were more prevalent in patients with an inadequate response to KD than in those with an adequate response (19). In these four studies, the epileptic symptoms were alleviated to different degrees after KD treatment, and GM composition and function changed to some extent after KD treatment.

**Table 2:** Summary of previous study on intestinal microbiota in epileptic patients with KD treatment.

Author	Patient Group	Age	Epilepsy Type	Intervention	Key Finding
Xie et al. (40)	EP (*n* = 14) *vs*. HC (*n* = 30)	EP: 1.95 ± 3.10 HC: aged up to 3 years	Refractory epilepsy	KD for 1 week	Healthy group: ↑Bacteroidetes;↑Actinobacteria Refractory group: ↑ Proteobacteria,↑Firmicutes KD treated group: ↑Bacteroidetes;↓Proteobacteria, *Cronobacter*
Zhang et al. (61)	EP (*n* = 20)	EP: median age is 4.3 years	Refractory epilepsy	KD for 6 months	After KD treatment: ↓Alpha diversity;↑Bacteroidetes,↓Firmicutes In the non-responsive group: specific gut microbiota is enriched
Lindefeldt et al. (62)	Children with epilepsy (*n* = 12) *vs.* parents (*n* = 11)	EP: 2–17 years	Refractory epilepsy	KD for 3 months	Parents group:↑Bacteroidetes, Proteobacteria;↓Actinobacteria, Firmicutes, After KD treatment: ↑Proteobacteria (*E. coli*);↓Actinobacteria, *Dialister*, *Bifidobacteria*, and *E. rectale;* Alpha diversity does not change significantly Function: Changes in 29 SEED subsystems
Gong et al. (63)	EP (*n* = 12) *vs.* HC (*n* = 12)	EP: 2–8 years	Refractory epilepsy	KD for 6 months	DR group: ↑Alpha diversity;↑Actinobacteria, *Enterococcus*, *Anaerostipes*, *Bifidobacterium*, *Bacteroides*, and *Blautia* After KD: ↑*Subdoligranulum*, *Dialister*, *Alloprevotella;* ↓*Bifidobacterium*, *Akkermansia*, *Enterococcaceae*, *Actinomyces* In the non-responsive group: some taxa are more prevalent

KD, ketogenic diet; EP, patients with epilepsy; HC, health control group.

Olson et al. studied two mouse models of refractory epilepsy and found that KD could increase the GABA/glutamate ratio in the colonic lumen, serum, and hippocampus by modulating key bacterial species, resulting in seizure reduction (20). This is the first study to verify the role of GM in the antiseizure effects of KD treatment in a mouse model. The protective effect of KD was abrogated in GF or Abx-treated SPF mice, while recolonization with KD-associated bacteria restored the epilepsy protection of KD treatment to normal levels (20). This phenomenon suggests the essential role of the GM in the epileptic protective mechanism mediated by KD. Further, this study revealed the possible underlying cellular and molecular pathways by which specific GM interact with each other to modulate peripheral metabolites and then impact the levels of hippocampal neurotransmitters (20). In a recent animal study, the abundance of Firmicutes was increased, and *Acetatifactor*, *Anaerotaenia*, *Escherichia*, *Flintibacter*, *Oscillibacter*, and *Erysipelatoclostridium* were higher in the KD group than in the ND group. The GM with increased abundance was related to the production of SCFAs and GABA (21). Although KD has specific value in the treatment of epilepsy and neurodegenerative diseases, it increases the risk of glucose and lipid metabolism disorders. The glucose intolerance and lipid accumulation induced by KD are closely related to the source and proportion of fat in the diet, which could be associated with alterations in GM composition (65).

There are still some questions on the mechanism whereby KD could protect epileptic patients from seizures. For example, how does modulation of bacterial species affect changes in membrane potential of hippocampal neurons? Is modulation of GABA/glutamate levels the main pathway? How can changes in bacterial species modulate GABA/glutamate levels? These questions should be the subject of future research.

## 4 Mechanism of the Correlation of Microbiota–Gut–Brain Axis and Epilepsy

### 4.1 Immune and Inflammation Pathways

The pathogenesis of epilepsy is linked to neuroimmunity and neuroinflammation (66). Accumulating evidence has demonstrated that immune and inflammatory pathways in the brain–gut axis may be involved in the pathogenesis of epilepsy. Microglia and astrocytes are the main inflammatory cells in the CNS, and their inflammatory state promotes the occurrence of epilepsy (67, 68).

#### 4.1.1 Gut Immunity

The lymphoid tissue of the intestinal mucosa contains 70%–80% of all immune cells in the body (69). The GM affects immune cells; for example, germ-free (GF) mice show immune abnormalities with a decreased population of T and B cells and a reduced cytokine production (70). Further, the GM appears as one of the most important factors for the maturation of microglial cells, as well as the astrocyte activation (71), which is age- and sex-dependent (72). The GM regulates innate immunity, adaptive immunity, and inflammatory mechanisms to modulate the development of epilepsy.

#### 4.1.2 Gut Barrier and Blood–Brain Barrier

The intestinal mucosal barrier and blood–brain barrier (BBB) work together to prevent GM and its secretions from entering the brain. “Leaky gut” syndrome is characterized by increased intestinal permeability, which allows bacteria, toxic metabolites, and small molecules to translocate into the bloodstream (73). Under gut inflammation, bacteria can directly release factors into the systemic circulation, which activates peripheral immune cells, alters BBB integrity and thus transport rates, and can even induce “leaky brain” (74). Stress can increase intestinal mucosal permeability, and lipopolysaccharides and other cytokines in the lumen enter the blood circulation stimulating toll-like receptors, producing inflammatory cytokines that could increase BBB permeability and damage the brain (75, 76).

#### 4.1.3 Neuroimmunity

Astrocytes are the most abundant glial cells in the brain and have a variety of functions, including regulating the integrity of the BBB, the recycling of neurotransmitters, and participating in immune responses (77). Microglial cells are the resident macrophages of the CNS, mediating the innate immune response (78). Microglia with a larger, less ramified, amoeboid morphology can promote inflammation, neuronal activity regulation, phagocytic neuron clearance, and chronic seizures (79). Microglia and astrocytes are involved in the pathogenesis of epilepsy by releasing excess cytokines (67), and interact with each other: microglia can modulate astrocytes’ phenotype and function (80), while mouse microglia can regulate astrocytes’ behavior through, for example, VEGF-B, which promotes the pathogenic response and inflammatory response of astrocytes, and TGF-α, which promotes the opposite (81). Gut microbes metabolize dietary tryptophan into aryl hydrocarbon receptor agonists and interact with its receptor to control microglial activation and TGF-α and VEGF-B expression, thereby modulating astrocyte pathogenic activity (81, 82). Inflammatory cytokines and chemokines released by astrocytes enhance microglial activities, including migration, phagocytosis of apoptotic cells, and synaptic pruning (83). The interaction between astrocytes and microglia leads to increased pro-inflammatory cytokine production and BBB permeability, which results in the infiltration of peripheral blood immune cells and cytokines into the CNS, and subsequent chronic neuroinflammation (84). GF and antibiotic-treated (Abx-treated) animals have also altered microglial morphology and defects in maturation, activation, and differentiation, resulting in an inadequate immune response to a variety of pathogens, which could be repaired after GM recolonization, which suggests that intestinal microbial diversity is critical for microglial and CNS function (85). In addition to glial cells residing in the CNS, peripheral immune cells, such as T cells and monocytes invading the brain tissue, are also involved in epilepsy’s morbidity. Monocytes can differentiate into macrophages and invade the brain, where they differentiate into “microglia-like cells” and contribute to epilepsy (86). As the organ with the largest population of immune cells, the gut is likely to play a role in this process, but the exact mechanism requires further investigation.

The GM can induce epilepsy through the innate immune pathway. BBB permeability increases throughout the life of GF mice related to the decreased expression of occludin and claudin-5 proteins in the endothelium (87). GM dysbiosis decreases claudin production and increases the permeability of the intestinal lining, leading to the escape of microorganisms, metabolites, and toxins from the intestinal lumen (88). GM dysbiosis also reduces SCFAs, which increases BBB permeability and promotes neuroinflammation (89). If these two barriers are broken, the immune cells and factors released by the microbiota enter the brain and induce seizures. Peptidoglycan (PGN) is a component of the bacterial cell wall that mainly exists in the human intestinal tract (90). PGN, as a driver of chronic encephalitis, has also been detected in brain microglia (90). Chronic inflammation, such as sclerosis-related temporal lobe epilepsy, is also a cause of epilepsy (91). Therefore, we conclude that PGN may translocate from the gut to the CNS by promoting gut leakage and brain leakage, leading to chronic inflammation and contributing to the occurrence of epilepsy.

The GM also contributes to epilepsy generation by inducing adaptive immunity. The GM can induce immune cells to produce cytokines that enter the brain through the intestinal mucosa and BBB and activate brain immune cells to participate in the immune response. T helper cell 17 (Th 17) cells are a proinflammatory CD4+ T cell subtype and key components of adaptive immunity (92). IL-17 is a cytokine produced by Th 17 cells, which can be modulated by specific GM phyla, such as Bacteroidetes (92, 93). As recently shown, both in the CSF and in the peripheral blood of patients with epilepsy, IL-17 levels were higher than in controls, and highly correlated with the frequency and severity of seizures (94–97). Therefore, the GM can influence the occurrence of epilepsy by mediating IL-17. GM metabolites, such as SCFAs, can affect the synthesis and secretion of immunoglobulins by regulating B lymphocyte differentiation (98, 99). The absence of commensal microbiota downregulates IgA and IgG1, and upregulates IgE, which leads to increased susceptibility to diseases (100, 101).

Therefore, GM can induce an immune response through the gut–brain axis, which leads to epileptogenesis. However, only a few studies have directly focused on the relationship between the gut, immune responses, and epilepsy, and many issues remain to be explored.

### 4.2 Nervous System

One of the most important pathways for transmitting information between the brain and the gut is *via* autonomic nerve fibers (102). Oral inoculation of *Campylobacter jejuni* to a mouse model leads to increased c-fos expression in the sensory ganglia and primary sensory relay nucleus of the vagus nerve in the brainstem, suggesting that gut stimulation can modulate brain activity *via* the autonomic nervous system (103). Vagus nerve stimulation (VNS) has become a normal therapy for epilepsy since being first reported in 1988 (104). Ressler et al. reported that electrical stimulation of vagal afferent fibers could modify brain concentrations of serotonin, GABA, and glutamate, thus explaining its use in epilepsy (105). VN goes through all intestinal layers, except the epithelial one, so it cannot directly interact with the GM (106). Enteroendocrine cells (EECs) can detect signals released from the luminal microbiota through different receptors. Previously, gut endocrine cells and the cranial nerve were thought to communicate only through hormones (107); however, Kaelberer et al. found that EECs named neuropod cells could synapse with vagal neurons to transduce gut luminal signals to connect the intestinal lumen to the brainstem using glutamate as a neurotransmitter (107). The discovery of neuropod cells provides a strong theoretical support for the treatment of neurological diseases by regulating the GM.

### 4.3 Enteroendocrine Signaling and Microbial Metabolites: Neurotransmitters and Short-Chain Fatty Acids

#### 4.3.1 Enteroendocrine Signaling and Neurotransmitters

Neurotransmitter imbalance is closely related to epilepsy. Neurotransmitter imbalance exists in epileptic foci, such as GABA with hypoactivity and glutamate with hyperactivity, dopamine and norepinephrine (NE) with hyperactivity, and serotonin with hypoactivity (108). In the gastrointestinal tract, neurotransmitters can be secreted directly by the GM or produced by gastrointestinal cells under the stimulation of GM metabolites. Different GM can produce different neurotransmitters (*Enterococcus* spp., *Streptococcus* spp., and *Escherichia* spp. produce serotonin; *Lactobacillus* spp. and *Bifidobacterium* spp. produce GABA, *Escherichia* spp. and *Bacillus* spp. produce NE and dopamine). The various neurotransmitters produced by the GM can pass through the intestinal mucosa but rarely through the BBB, with the exception of GABA (109). In hippocampal injury, or epileptic status, GABA produced by GM can lead to an imbalance between the GABA and glutamate systems, causing seizures. Sun et al. showed that the relative abundance of the genera *Coprococcus, Ruminococcus*, and *Turicibacter* was positively correlated with glutamate and glutamine levels (110). The GM can affect the glutamine–glutamate–GABA cycle, produce neurotransmitters, and mediate the expression of GABA and NMDA receptors in specific brain regions (hippocampus, amygdala, and locus coeruleus) (111). *A. mucinophilia* and *Parabacteroides* colonization could alter the level of amino acids in the serum and gut lumen to modulate the levels of seizure-associated neurotransmitters, such as GABA and glutamate, in the hippocampus, thus providing protective anti-seizure effects in mice (20, 112). Enterochromaffin cells (ECs) produce approximately 90% of 5-hydroxytryptamine (5-HT) (113). In GF mice, certain intestinal microbiota, such as spore-forming clostridia taxa, can promote 5-HT biosynthesis in the gut by upregulating colonic tryptophan hydroxylase 1, a rate-limiting enzyme for 5-HT production (114, 115). Previous studies have shown that patients with temporal lobe epilepsy have a 5-HT deficiency. A drug combination that increases 5-HT, such as selective serotonin reuptake inhibitors, may improve seizure control in patients with epilepsy (116). The 5-HT decrease induced by reserpine appears to increase susceptibility to minimal electroshock seizures in rats (117). However, changes in intestinal 5-HT levels could not directly affect the brain, as 5-HT cannot cross the BBB (118). Chemotherapeutic drugs often cause nausea and vomiting, caused by the release of large amounts of 5-HT in the intestine and the subsequent activation of vagal afferents (119). 5-HT released by ECs may have a potential impact on brain–gut axis signal transduction by regulating intestinal vagal afferent activity (120) and inflammatory responses (121). Alterations in 5-HT signaling are associated with irritable bowel syndrome (122). Therefore, we speculate that a change in 5-HT levels in the intestine may be related to epilepsy, but there is no evidence to support this. N-acetyl aspartic acid (NAA) levels are reduced in patients with epilepsy, and in the epileptic suckling pig model, Austin et al. found that low NAA levels were associated with fecal *Ruminococcus*, and this process may be mediated by serum cortisol (123). NE has a double effect on epilepsy onset depending on its amount, NE at low doses has pro-epileptic effects, while high doses could inhibit epilepsy (124). Dopamine, serotonin, and acetylcholine are closely correlated with epilepsy and could indirectly affect brain function through the enteric nervous system, the vagus nervous system, and by regulating the expression of peripheral receptors (125).

#### 4.3.2 SCFAs

SCFAs, including acetate, propionate, and butyrate, can be produced by some gut bacteria (mainly Bacteroides and Firmicutes) through the fermentation of insoluble dietary fibers (126). SCFAs play an essential role in microglial maturation, the gut–brain nervous system, BBB permeability, and stress responses through direct or indirect pathways, all closely related to epilepsy (127). As mentioned earlier, SCFAs were reduced in WAG/Rij rats, and butyrate had an anti-absence seizure effect (16). The protective effects and mechanisms of different SCFAs in epilepsy were further studied in a PTZ-induced epileptic mouse model (8, 128, 129). Sodium butyrate may exhibit antiepileptic effects in PTZ-induced epileptic mice by alleviating intestinal inflammation and oxidative stress (8). Butyrate also ameliorates mitochondrial dysfunction and protects brain tissue from oxidative stress and neuronal apoptosis through the Keap/Nrf2/HO-1 pathway, thereby increasing seizure threshold and reducing seizure intensity (129). Propionate treatment may alleviate seizure intensity and prolong the incubation period of seizures by reducing mitochondrial damage, hippocampal apoptosis, and neurological deficits (128). These studies show that SCFAs are reduced in epilepsy models and their protective effect on epilepsy through different mechanisms.

### 4.4 The HPA Axis

Stress can promote the induction of epilepsy, and epileptic patients have higher glucocorticoid levels (130). The HPA axis is central to stress responses, including the secretion of corticotrophin-releasing factor, adrenocorticotropic hormone, and subsequent release of glucocorticoids (e.g., cortisol, corticosterone, deoxycorticosterone, and corticotrophin) and catecholamine downstream pathways (131). The HPA axis is regulated either by the negative feedback of glucocorticoids or by input from numerous different brain regions, including the prefrontal cortex, hippocampus, amygdala, and the bed nucleus of the stria terminalis (132). Different hormones may have different effects; for example, most deoxycorticosterones are anticonvulsants, whereas corticotropin-releasing hormone and corticosterone could induce seizure activity (133, 134). Despite the correlation between the HPA axis and GM, the specific mechanism has not been elucidated (135). Chronic stress could upregulate glucocorticoids, which could enhance glutamatergic signaling and induce seizures (136). GM can affect the function of the hypothalamus by changing circulating cytokine levels or other pathways, thereby regulating the HPA axis (137). Stress responses in both SPF and GF mice suggest that the GM modulates stress-dependent pituitary and adrenal activation and alters the expression of genes regulating the corticotropin-releasing hormone pathway in the colon (138). We hypothesized that chronic stress may affect the HPA axis through the GM and promote epilepsy; however, the specific relationship among the HPA axis, GM, and epilepsy still needs to be further investigated.

## 5 Brain–Gut Axis as Potential Diagnostic and Therapeutic Target for Epilepsy

### 5.1 The GM Perspective in Differential Diagnosis of Epilepsy

The GM differences between healthy people and patients with different types of epilepsy make it a potential biomarker for differential diagnosis, prognosis, and treatment monitoring in epilepsy. Fecal microbiota may not accurately reflect GM situation because of possible pollution, and although colonoscopy is more accurate than fecal GM, its invasiveness limits its clinical application. In addition, the GM is affected by age, diet, living environment, and other factors, the sample size in these studies was small, and there were some contradictory results. Therefore, the GM needs to be further studied in larger patient samples and under strictly controlled variables.

### 5.2 The Value of the Brain–Gut Axis in the Treatment of Epilepsy

**Figure 2:**
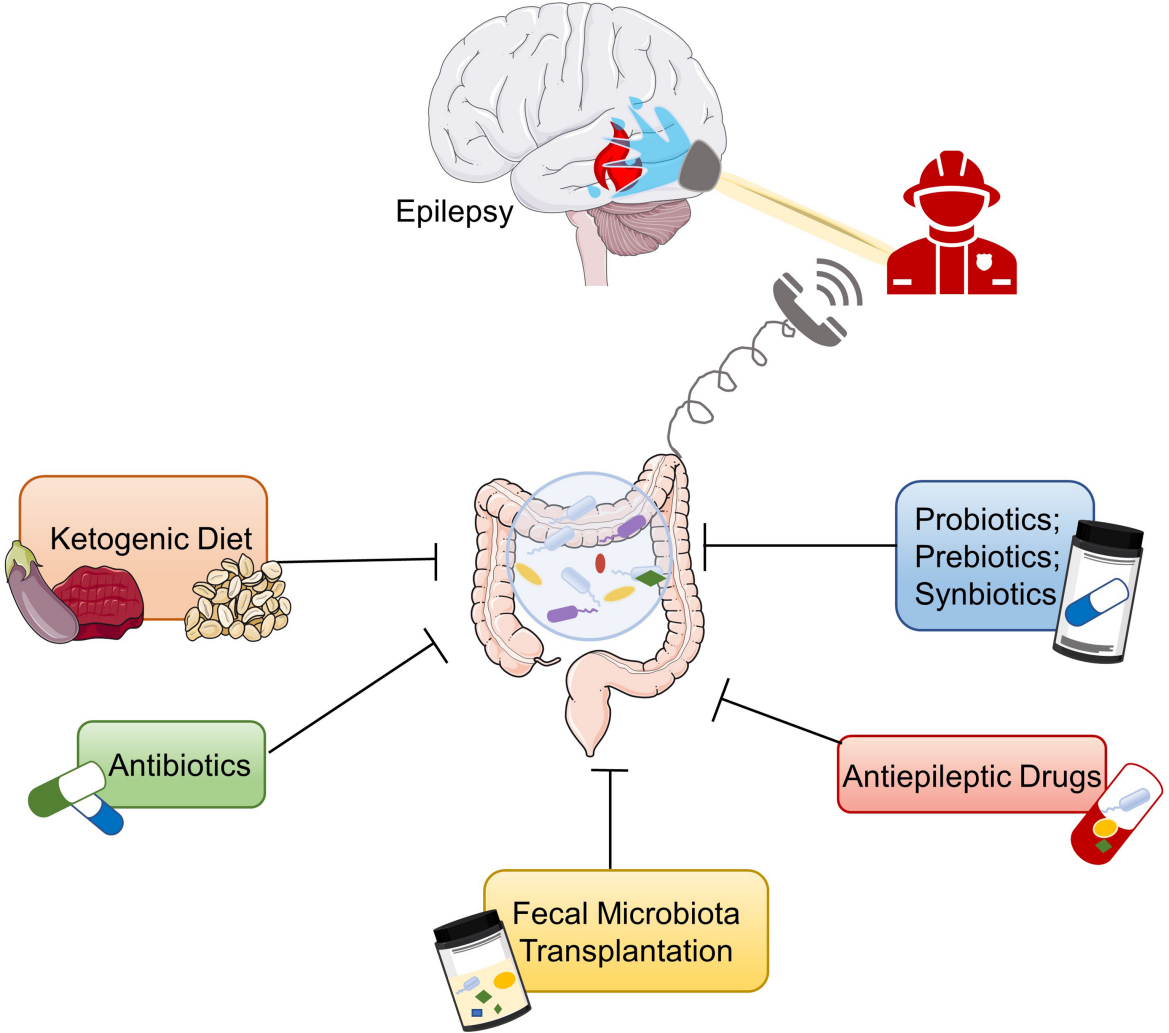
Potential therapies for epilepsy based on gut microbiota. Ketogenic diet, antiepileptic drugs, probiotics, prebiotics, synbiotics, antibiotics, and fecal microbiota transplantation are potential treatments for epilepsy based on the microbiota–gut–brain axis.

Modulation of the GM may be a potential therapeutic approach for intractable epilepsy. On the one hand, regulation of GM could reduce the occurrence of seizures by adjusting the mechanisms related to epilepsy. On the other hand, drugs can be converted into metabolites by GM through direct or indirect ways to exert therapeutic efficacy or cause toxic side effects (139). For DRE patients, adjusting the composition of the gut microbiome may promote drug metabolism and absorption and increase their responsiveness to antiepileptic drugs. In this section, we will review the effects of dietary intervention, antibiotics, probiotics, prebiotics, synbiotics, antiepileptic drugs, and fecal transplantation on epilepsy (**Figure 2**).

#### 5.2.1 Dietary Intervention

Diet, especially KD, could regulate the occurrence of epilepsy by shaping GM as discussed in detail in *Section 3*. KD is a high-fat, low-carbohydrate, and adequate protein diet used since 1921 in patients with refractory epilepsy (140). KD also has positive effects on other neurological diseases, such as multiple sclerosis, Parkinson’s disease, Alzheimer’s disease, and migraine (141–143). The classic ratio of fat to protein and carbohydrates in KD is 4:1 (18), which triggers a metabolic pattern shift from glucose metabolism toward the metabolism of fatty acids (53). The classic KD could relieve epilepsy by multiple pathways, including modulation of neurotransmitters, brain energy metabolism, oxidative stress, ion channels, and GM (53, 54, 144).

In addition to the classical form, several modified KD diets have arisen, including the modified Atkins diet, medium-chain triglyceride diet, low-glycemic index treatment, and modified Mediterranean ketogenic diet (MMKD), with various composition ratios of fat, protein, and carbohydrates (145). In medium-chain triglyceride diet, medium-chain triglycerides are used instead of long-chain triglycerides (145). MMKD is characterized by olive oil as a source of monounsaturated fatty acids (146). Olive oil contains antioxidant molecules such as monounsaturated fatty acids and polyphenols, which have beneficial effects on inflammation, cardiovascular disease, and oxidative status of the body (147). MMKD can regulate the GM of patients with mild cognitive impairment, especially fungal flora (146). A 12-month KD based on olive oil could alleviate symptoms in 83.1% of patients with refractory epilepsy, comparable to the effect of a traditional KD treatment (147). However, there is no comparative study between MMKD and traditional KD on the efficacy and side effects on refractory epilepsy. At present, most KDs are individualized modified KDs balanced between ketogenic effect and palatability.

In KD, saturated fat has always been the prominent fat used (148); however, animal and human studies have demonstrated the anti-epileptic effects of polyunsaturated fatty acids (PUFAs), especially omega-3(n-3) PUFAs (149). Dietary n-3 PUFAs are found in flaxseed, nuts, marine fish, and marine mammals. N-6 PUFAs are mainly derived from animal products and vegetable oils and constitute the majority of PUFAs in the modern Western diet (149). Docosahexaenoic acid (DHA, 22:6n-3), the primary n-3 PUFA in the brain, participates in the regulation of neural function through a variety of pathways, such as interaction with ion channels and neurotransmitter release (150, 151). A case–control study indicated a lower serum omega 3/omega 6 ratio in children with epilepsy than in healthy children (152). Both *in vitro* and *in vivo* studies demonstrated that a diet rich in n-3 fatty acids is beneficial for epilepsy control, but the results of clinical studies are somewhat contradictory (149). A meta-analysis of seven clinical trial studies in 2021 indicated that omega-3 supplementation significantly reduced seizure frequency and was more effective in adults than in children (153). Therefore, adjustment of dietary n-3/n-6 levels could be associated with seizure control. A recent study indicated that a high [fat]:[carbohydrate + protein] ratio is not indispensable for the treatment of epilepsy (154). A new combined diet with low [fat]:[proteins + carbohydrates] ratio, including medium-chain triglycerides, PUFAs, low glycemic index carbohydrates, and a high branched-chain amino acids/aromatic amino acids ratio also reduces excitatory drive and protects against seizures in rodent models (154). Although only tested on animals, it is a promising diet with fewer side effects.

Dietary intervention is an effective and perspective way to control epilepsy, and further research on the bacterial–gut–brain axis would contribute to develop the more effective dietotherapy.

#### 5.2.2 Probiotics/Prebiotics/Synbiotics

Probiotics are living microorganisms that, at the appropriate dose, are beneficial to the host health (155). Most common probiotics include *Bifidobacterium* and *Lactobacillus* (156). In 2020, the International Scientific Association for Probiotics and Prebiotics defined prebiotics as a “substrate selectively utilized by host microorganisms conferring a health benefit” (157). Synbiotics has been defined as “a mixture comprising live microorganisms and substrate(s) selectively utilized by host microorganisms that confers a health benefit on the host” consisting of two subsets: synergistic synbiotic (the prebiotic is selectively utilized by the co-administered live microorganisms) and complementary synbiotic (each component works independently) (157). In a prospective study, the frequency of seizures was reduced by ≥50% in 28.9% of DRE patients treated with a probiotic mixture for 4 months, and 76.9% of these improved patients maintained a lower seizure frequency 4 months after discontinuation (158). This study indicated that adjuvant probiotics reduced the frequency of seizures and could be used as a complementary treatment to antiepileptic therapy (158). In the PTZ-induced chemical kindling mouse model, the probiotic supplementation group did not show full kindling, and GABA increased in mouse brain tissue, which indicated that probiotic supplementation could substantially reduce seizure severity (159).When treating PTZ-induced seizures in mice with KD, synbiotics or *Lactobacillus fermentum* MSK 408 could reduce the side effects of KD without disturbing its antiepileptic effects (21, 160). Both KD and MSK 408 increase GABA metabolism by regulating the GM (160). Several SCFAs, such as propionate and butyrate, have antiepileptic effects. After a month of intervention with the classic KD, the total SCFAs were significantly reduced, especially acetate, propionate, and butyrate, which may be due to a reduction in the intake of fermentable carbohydrates or a reduction in fermenting bacteria by KD (161). Synbiotics can lead to GM enrichment associated with SCFAs (21). MSK 408 could influence SCFAs and restore serum lipid profile and tight junction protein mRNA expression in the gut and brain independently by modulating the GM (160).

These studies are preliminary observations of supplementary probiotics in the treatment of DRE, and further theoretical validation and mechanism exploration in larger placebo-controlled trials and more rigorous animal experiments should be conducted. Probiotics have the potential to be a complementary treatment for refractory epilepsy and can be used in combination with KD therapy to reduce side effects.

#### 5.2.3 Antibiotics

Antibiotics can have either good or negative effects on the treatment of inflammatory bowel disease, irritable bowel syndrome, hepatic encephalopathy, and other diseases (162). Despite the little evidence on the link between the use of antibiotics and seizures, a retrospective study of six refractory epileptic patients treated with antibiotics showed that certain antibiotics could reduce the frequency of seizures in the short term (163). They hypothesized that antibiotics might induce seizure freedom or decrease seizure frequency by interfering with the intestinal flora and the gut–brain axis (163). However, certain antibiotics can also induce epilepsy; for example, lactam antibiotics, including penicillin, cephalosporins, and carbapenems, are most likely to cause seizures (164). Unsubstituted penicillin, like fourth-generation cephalosporins, imipenem, and ciprofloxacin in combination with renal dysfunction, brain lesions, and epilepsy could lead to an increased risk of symptomatic seizures. Therefore, serum levels and EEG should be closely monitored when using antibiotics in these patients (165). Antibiotic application exerts short- or long-term effects on GM composition in both humans and animals (162). Although some antibiotics can disrupt the balance of intestinal microorganisms and cause diseases, others increase the abundance of beneficial microorganisms and play a positive role in the GM (162). Different antibiotics lead to different patterns of GM alteration; for example, macrolides induce the reduction of Actinobacteria (mainly *Bifidobacteria*) (166, 167), oral vancomycin reduces Firmicutes and increases Proteobacteria (168), whereas penicillin only has a weak effect on the human microbiota (168). The extent of amoxicillin-induced epilepsy is not parallel to GM changes, which is contradictory to the hypothesis that GM acts as a bridge in antibiotic-induced epilepsy. However, the influence of antibiotics on GM is also related to the initial GM composition (169) and habits of the host (170). In the future, multi-center cooperation is needed to further clarify the specific effects and mechanisms of various antibiotics on epilepsy.

#### 5.2.4 Antiepileptic Drugs and GM

The GM contains a rich variety of drug-metabolizing enzymes that influence their pharmacology, resulting in interpersonal differences in drug efficacy and toxicity (171). For example, clonazepam is an anticonvulsant and anti-anxiety drug reduced and metabolized by the GM, resulting in drug toxicity (172). Non-antibiotic drugs alter the GM to some extent. In a large study involving the effects of 1,197 non-antibiotic drugs on 40 GM, 24% of the drugs with human targets inhibited the growth of ≥1 strain *in vitro* (173). Antiepileptic drugs, such as carbamazepine, valproic acid, and lamotrigine affect GM composition (174, 175). Valproic acid treatment during pregnancy in mice resulted in altered fecal microbiota (176, 177), with increased Firmicutes and decreased Bacteroidetes (178), which may be associated with ASD-like behavior in offspring (176, 177). Lamotrigine might decrease the growth of *E. coli* by inhibiting bacterial ribosome biogenesis (179). Further research on the relationship between antiepileptic drugs and gut microorganisms will help to develop new antiepileptic drugs based on the principle of GM regulation. Adjusting GM composition could alter the metabolic process of antiepileptic drugs to improve their efficacy and reduce side effects.

#### 5.2.5 FMT

FMT has been proven to be an extremely effective treatment for recurrent or refractory *Clostridium difficile* infections (176, 177, 180, 181). Moreover, FMT has been extensively studied as a potential treatment for GM-related diseases. Its effectiveness has been demonstrated in a range of diseases, such as ulcerative colitis (182), hepatic encephalopathy (183), irritable bowel syndrome (184), obesity (185), and even neurological disorders (186, 187). Recent studies have revealed a correlation between epilepsy and GM; thus, the value of FMT administration in patients with epilepsy has been further investigated. FMT has been shown to prevent the recurrence of epilepsy after discontinuation of antiepileptic drugs in patients with Crohn’s disease and a long history of epilepsy (188). In a rat model of epilepsy with basolateral amygdala kindling following chronic stress, GM transplantation from chronically stressed rats to sham-stressed rats accelerated the kindling epileptogenesis process, whereas transplantation from sham-stressed group to stressed rats reduced the pro-epileptic effects of stress (42). Olson et al. observed an increased seizure threshold in a GF temporal lobe epilepsy mouse model after transplantation with microbiota from KD-treated mice or long-term administration of microbiota associated with KD treatment (20). However, there are still some challenges to the FMT process. Currently, FMT is performed by placing small amounts of liquefied or filtered feces directly into the colon or through a feeding tube, enema, or capsule (189). Therefore, FMT may result in the transmission of bacteria, viruses, or diseases that cannot be detected by screening (189, 190). Furthermore, FMT may disrupt the baseline microbiota diversity, resulting in the breakdown of colonization resistance to a broad spectrum of harmful microorganisms (190). Therefore, more long-term follow-up studies are needed to determine the efficacy and safety of FMT in patients with epilepsy before large-scale clinical application.

## 6 Gut–Brain Psychology and Epilepsy

In addition to epilepsy, current research have linked the brain–gut axis to the development of many other neuropsychiatric disorders, such as neurodegenerative diseases and mental disorders (191, 192). Psychiatric diseases are often comorbid with epilepsy (193, 194). Patients with epilepsy have an increased risk of mental illness, which increases their disability and mortality rates (193, 194). Depression is the most common comorbidity in patients with epilepsy (195). Patients with epilepsy have a twofold increased risk of depression compared to the standard population (196). Mental disorders, such as depression, anxiety, dipolar disorder, and schizophrenia, could be rooted in abnormal GM (192, 197). Though gut–brain axis in psychology has been rapidly developed over the decades (192), the conception of “gut–brain psychology” was firstly proposed by Jin et al. in 2018: the discipline of studying the relationship between the gut–brain and mind (192). Neuroinflammation, HPA axis hyperactivity, and altered neurotransmitters (such as 5-HT) are common mechanisms for epilepsy and comorbid depression, and involved in the gut–brain axis (117, 198, 199). KD, a well-known treatment for epilepsy, has been shown to play a role in psychiatric disorders (200). Other than KD, antibiotics, FMT, probiotics, and prebiotics also have potential in regulating both mood and epilepsy (158, 163, 188, 192). Therefore, in the process of studying the relationship between epilepsy and brain–gut axis, the gut–brain psychology should be considered. In the future, it may be possible to treat neuropsychiatric diseases and improve brain and mental health by manipulating the microbiome and the gut–brain axis.

## Conclusion

Advances in sequencing techniques have deepened our understanding of GM, and an increasing number of studies have indicated the indispensable role that the brain–gut axis plays in epilepsy. The autonomic nervous system, enteric nervous system, neuroendocrine system, and neuroimmune system all contribute to communication between the brain and the gut. KD has been long used in the clinical practice as an effective treatment for intractable epilepsy. The involvement of the brain–gut axis further clarifies the potential mechanism behind this treatment method. In addition, controlling GM by probiotics, prebiotics, synbiotics, antibiotics, or FMT could reduce the frequency of seizures and improve the threshold of epilepsy. Gut–brain psychology should be considered in the study of gut–brain axis in epilepsy. At present, research on the relationship between the brain–gut axis and epilepsy is still at the preliminary stage. Future research will help identify new diagnostic and therapeutic targets for refractory epilepsy in relation with the gut–brain axis.

## Author Contributions

MD and YL conceived the topic and designed the outline of this review. MD, YL, HS, and JS contributed to the manuscript writing. MD prepared the figures and tables. LC critically revised the manuscript. All authors contributed to the article and approved the submitted version.

## Funding

This work was supported by a grant from the National Natural Science Foundation of China (82071351).

## Conflict of Interest

The authors declare that the research was conducted in the absence of any commercial or financial relationships that could be construed as a potential conflict of interest.

## Publisher’s Note

All claims expressed in this article are solely those of the authors and do not necessarily represent those of their affiliated organizations, or those of the publisher, the editors and the reviewers. Any product that may be evaluated in this article, or claim that may be made by its manufacturer, is not guaranteed or endorsed by the publisher.
